# How to avoid pitfalls in antibody use

**DOI:** 10.12688/f1000research.6894.1

**Published:** 2015-09-07

**Authors:** Diana Pauly, Katja Hanack

**Affiliations:** 1 Department of Ophthalmology, University Hospital Regensburg, Regensburg, 93053, Germany; 2 Department of Immunotechnology, University of Potsdam, Potsdam, 14476, Germany

**Keywords:** antibodies, validation, characterization, target, application, databases, unique identifier

## Abstract

Antibody use is ubiquitous in the biomedical sciences. However, determining best research practices has not been trivial. Many commercially available antibodies and antibody-conjugates are poorly characterized and lack proper validation. Uncritical application of such useless tools has contributed to the reproducibility crisis in biomedical research. Despite early initiatives such as MIAPAR or PSI-PAR, a best practice guideline for antibody characterization is still not in prospect. Here, we analyze 24 antibody-related databases and compare their content with regard to validation aspects and coverage. We also provide a flowchart for end-users with all necessary steps to facilitate finding and choosing specific and sensitive antibodies for their experiments. Based on a growing demand for better and standardized validation procedures and characterization guidelines for antibody molecules we have summarized our findings in a five-point plan. We intend to keep the discussion alive and hope that properly used antibodies will remain as central to biomedicine as they are today.

Antibodies are ubiquitous tools in biomedical research to characterize and study proteins, protein-protein or protein-DNA interactions. Theoretically, they can be applied in almost every field but practically it is not as straightforward as one might naively expect. Although a lot of money has been invested and the global research antibody market is estimated to reach 2.64 billion Euros by 2019 ^1^, many antibody-related products still face the problem that they are not properly validated and/or critical experimental data are not accessible. This leads - justifiably - to a growing unease among researchers, evidenced in several recent high-profile publications regarding the lack of standardization, validation and reference of those crucial research tools ^2– 5^. Antibody-related products are often expensive and fail to meet the customers’ expectations. We have now more than ten years of experience in generating monoclonal antibodies and we are well aware of the difficulties and obstacles on the way to highly effective antibodies ^6– 11^. On the one hand, we realize that end-users expect to obtain highly specific tools. On the other hand, the potential use-cases of antibody technology are too broad for a simplistic order-and-use scenario. Antibody-use and especially antibody generation are complex procedures that are liable to both false-positives as well as false-negatives, if not carried out appropriately. The following questions should be at the starting point of any such project:

1. What is the target protein I want to characterize?
2. What is the application in which the antibody should work?
3. Which samples will be tested (serum, tissue, cells)? How is my target protein structured in these samples?

We have compiled a flow diagram for end users and manufacturers in Figure 1, which outlines the entire procedure. Already the step, finding the right antibody in multiple databases, is like looking for the proverbial needle in a haystack. Only by sheer luck or with an efficient strategy is it possible to find your antibody of choice in one of the over 24 antibody databases ( Supplementary file 1). An inordinate amount of time has to be invested to extract the important characteristics of an antibody from a jumble of detailed and often unreferenced information in these databases. There are a few specialized sites which can help in some well-defined research areas, like the *Antibody Validation Database*, the *ENCODE project* or the *San-Diego Epigenome Center*, which focus on histone modifications or the *Office of Cancer Clinical Proteomics Research Antibody Portal* and the *Abminer* website, which concentrate on antibodies specific for cancer-associated proteins and tissues. For essentially all other antibodies, filtering information in databases with millions and millions of antibody products is a great challenge. The most useful databases are those which include independent antibody results either by citing published research reports or by including user reviews. Ten out of the 24 websites we investigated include antibody-related publications in their result screens and only 7 offer an easy to find platform for user reviews or comments ( Supplementary file 1). Moreover, any of these two functionalities ideally ought to be combined with an option for comparison (available in 8 of 24 databases). There are additional unique features of individual databases which facilitate the search, including credits for user-reviews (e.g. *1DegreeBio, pAbmAbs, Biocompare*), rating systems (e.g. *Antibody-Adviser, 1DegreeBio, Biocompare, pAbmAbs, CiteAb, AntibodyReview*), special initiatives for independent validation (e.g. *Antibodies Online, Antibody Validation Database, Antibodypedia, The human protein atlas*) and direct ordering from the database (e.g. *Developmental Studies Hybridoma Bank, Antibodies Online*). However, sometimes it seems easier to hire a detective than to order a specific antibody.

In many cases, we do not succeed in tracking down the right antibody required for our project ( Figure 1). In these cases, we resort to project-specific antibody-generation based on monoclonal and polyclonal antibodies. In the planning stage, we place particular emphasis on the characterization of the target antigen and possible epitopes useful for immunization. This is necessary in order to find surface-related sequences available for antibody binding and to minimize possible cross reactivities of the antibody. Of course we agree with James Trimmer that “antibodies are not magic reagents” ^5^, but properly designed, characterized, validated and used, some can come close.

**Figure 1.**
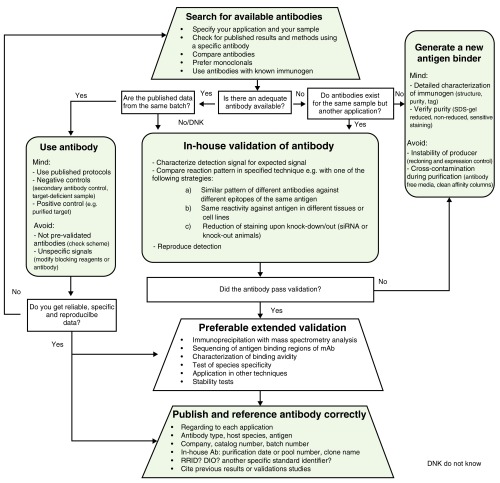
Step-by-step guide on how to identify and validate your antibody of choice.

An antibody can only bind the target used during immunization. The decision about the immunization strategy is all too often made without end-user input. Therefore, we would like to remind commercial producers of antibodies of their responsibility and support the growing demand for better validation and standardization tools ^12^ ( Figure 1). Therefore, we urge the community to revitalize the groundbreaking standardization ambitions of 2010 and revise the “Minimum information about a protein affinity reagent” (MIAPAR) and “Proteomics Standards Initiative-Protein affinity binders” (PSI-PAR) towards a simplified, common guideline for usage of affinity binders ^13, 14^.

We strongly disagree with the statements by Bradbury and Plückthun (2015) that polyclonal and hybridoma-generated monoclonal antibodies should be discarded from the biomedical research portfolio ^2^. We also decline the exclusive value of recombinant antibodies. The disadvantages of polyclonal sera and monoclonal antibodies can be minimized by proper research practices ( Table 1), such that they are far outweighed by the advantages. It is impossible to deny that sequencing antibodies is helpful in order to reliably produce them recombinantly. The main problem, however, is not the lack of sequence data but the absence of standardized assessments of antigen binding. In most common use cases, with proper research practices, sequencing antibodies becomes a matter of convenience rather than necessity.

**Table 1.** Disadvantages of monoclonal and polyclonal antibodies and the solutions.

**Problems with**	Solution
**Monoclonal antibodies**	
Instability of hybridoma cell lines	Quality process control including recloning and periodical intracellular immunoglobulin staining
Death of cell lines or loss of antibody genes	Sequencing of antibody genes and recombinant expression
**Polyclonal antibodies**	
Batch-to-batch variability	Correct reference in publication!; include at least company, catalogue number, batch number; if the antibody is house-made include bleeding date or pool number
Bind multiple targets	Careful characterization, immunoaffinity enrichment

Further we are convinced that there is an urgent need for proper identification of antibodies in order to avoid irreproducibility of research results and confusion of product similarities by rebranding of single antibodies. Sequencing of antigen-binding subunits is only one solution to add a unique, persistent identifier to each of these binders. Other initiatives, like the Encode accession number or Research Resource Identifier (RRID) will also help to identify existing antibodies in published reports ^15, 16^. In general, it should be the aim of the research community to prevent balkanization also of the persistent identifiers of antibodies and agree on a single identifier system with open standards. We are very interested in passing on our experience in antibody generation in order to create better standardization and validation workflows.

Addressing all the identified problems in the antibody field, we suggest a 5-point plan:

1. Combine all information about available antibodies in one comprehensive repository.
2. Standardize antibody validation.
3. Standardize antibody reference specifications in publications and add a unique identifier to each reagent.
4. Sequence important and relevant antibodies for future reliable use.
5. Generate specific, reliable and consistent binders for missing antigens using all techniques available.

## Supplementary materials

**Supplementary file 1: Antibody search websites.**

abbreviations: Ab antibody, WB Western Blots, ELISA enzyme-linked immunosorbent assay, IHC immunohistochemistry, FACS fluorescence-activated cell sorting, IF immunofluorescent, IP immunoprecipitation, DB dot blot, ChIP chromatin immunoprecipitation, siRNA small interfering RNA, CRISPR clustered regularly interspaced short palindromic repeats, shRNA small hairpin RNA, Immuno-MS immunoprecipitation with mass spectrometry analysis, SPR surface plasmon resonance spectroscopy, NAPPA nucleic acid programmable protein array, EM electron microscopy ^15, 17– 20^.
