# Supplementary material for: How to avoid pitfalls in antibody use
Source: F1000Res. 2015 Sep 7;4:691. [Version 1] doi: 10.12688/f1000research.6894.1 (PMC4722690; doi:10.12688/f1000research.6894.1)
Supplement: Supplementary file 1 [file f1000research-4-7423-s0000.tgz › fece9b05-2956-4c8a-974c-e841eaac793b.pdf]

Name	Link	Main focus	Ab	Targets	Target species	Sources	Independent validation	Published References	Vendor Validation	Applications	Compa-rison	RRID	Remarks	Ref.
**1DegreeBio**	http://1degreebio.org	"fast way to find reagents"	>100		31	17	x			24 different techniques		No	earn DB$ for participation, user ratings	
**Abminer**	http://discover.nci.nih.gov/abminer/	human cancer tissue	661		human		x			WB bands and dilution described, no pictures shown	x	No	Entrez Gene ID search possible	17
**Antibodies Online**	http://www.antibodies-online.com/antibody/	database with detailed information	944,908	<1000		137	x	x	x	WB, ELISA, IHC, FACS, IF, IP, DB, functional studies, neutralization and many others	x	No	antibodies have to be directly ordered from this database	
**Antibody adviser**	http://www.antibody-adviser.org/	all available antibodies are reviewed by users	356,526	33,401	57	97	x			all antibody-associated techiques		Yes	personal digital antibody logbook, always will stay free, non-profit-organization, reviews are revised	
**Antibody Validation Database**	http://compbio.med.harvard.edu/antibodies	histon modifications	261	65	Drosophila melanogaster, Caenorhabditis elegans and human cells	13	15 different validators			WB, DB, ChIP-chip/seq		No	25% of the antibodies failed validation process	18
**Antibodypedia**	http://www.antibodypedia.com/	"the database is gene-centric, validations and citations give a score and rank to the antibody"	1.8 M	94% of human genome (20507)	human	63	x	x	x	WB, IHC, Protein array, Proximity ligation assay, Reverse Phase Protein Arrays, Knock-down included from end of June and many others	x	Not, at the moment	new initiative called the Affinity Binder Knock-Down Initiative: confirmation an antibody binding to a target by means of knock-down (e.g. siRNA, CRISPR).	19
**AntibodyRegistry (see also Scicrunch)**	http://antibodyregistry.org/search (https://scicrunch.org/resources/Antibodies/search)	"Ab for all areas, stable identifying information for all Ab reagents ever sold"	2.4 M	>20000	any	>200		x				Yes, generate RRID	We do not remove records because it is our mission to keep a version of the antibody record even if the supplier or vendor does not keep the listing	
**AntibodyResource**	http://www.antibodyresource.com/	"Ab for all areas"	1.5 M	30000	23	100			x	FACS, WB, ELISA, IF, IHC and others		No	50,000 visitors per month and researchers can filter antibodies for multiple applications and species	
**AntibodyReview**	http://www.antibodyreview.com/index.php	"Ab against human proteins (incl. mouse and rat orthologues), literature search based"	540,000	~20000	mainly human, mouse, rat (>20 species)	55		x	x	WB, IHC, ICC, IP, FACS, ELISA and others		not currently, earlier efforts	unique scoring system, visual antibody search system by data images	
**Biocompare**	http://www.biocompare.com/Antibodies/				>30		x		x	WB, ELISA FACS, IHC, IP, functional assays and others	x	No		
**CiteAb**	http://www.citeab.com/	"aims to be the most comprehensive antibody search engine in the world"	2.15 M	~30.000	~1600 species / groups	119		x		Top applications listed:WB, IHC, ELISA, FACS		not currently	Search results are ranked by how frequently antibodies have been cited in the academic literature. (660,000 citations)	
**Developmental Studies Hybridoma Bank (DSHAB)**	http://dshb.biology.uiowa.edu/	monoclonals for research	2,655	1505		284 researcher		x				No	hybridoma cells by cost, non-profit	20

**ENCODE**	https://www.encodeproject.org/search/?type=antibody_lot	histone modifications	1,118 (diff. lots), 978 (unique Ab)	778	9	45	independly by different ENCODE members			IP, WB, ChIP-seq, DB, Knockdown/- out, motif enrichment, IF, ELISA, competetive assay		DOI or RRID will be explored in the future	color code for eligibility status, only 393 of 1118 Ab are useful for specific IP experiments, antibody lot specific data	15
**F1000-Antibody validation collection**	http://f1000research.com/channels/antibody-validation	publication of antibody validation reports, negative results are also published	19	9	human, mouse	9	x	x		WB, shRNA-mediated knock-down, IHC		Yes	detailed protocols for antibody use, includes pAbmAbs rating	
**iSpyBio**	http://www.ispybio.com/	"give as large a selection as possible, so it does not focus on a specific area"	1.3 M	13,000	all species		x	x	x	WB, ELISA, IHC, ChIP and others	x	No	"ranks products based on publications, application data, reviews and images"	
**Labome**	http://www.labome.com/		>42,705	>8036				x	x	WB, IHC, FACS, IP, ChIP, ELISA and others		No	publish antibody reviews, curate antibody information in PLOS Biology and eLife	
**Linscott`s Directory**	http://www.linscottsdirectory.com/	since 1980 summary of products for immunological and bioloigcal reagent	1.29 M			139						No	direct link to companies	
**Office of Cancer Clinical Proteomics Research Antibody Portal**	http://antibodies.cancer.gov/apps/site/default	cancer related proteins	328	147			7 expert organisations			ELISA, WB, IHC, Immuno-MS, SPR, NAPPA		No	distribution by DSHAB (non-profit) and companies	
**Open i**	http://openi.nlm.nih.gov/	image search machine in literature	all published with figures	all published with figures	all published with figures	all published with figures		x		all published with figure		depends on journal	search for published figures, check for antibody in material and method section	
**pAbmAbs**	www.pabmabs.com	"searchable database of independent antibody reviews from scientists"	1000			60	x			all possible techniques, e.g. WB, IHC, IP, ELISA, FACS, Luminex, EM		No, will be included in the future	application-specific star rating system based on a Bayesian algorithm, pAbMAbs competition for the best submitted review	
**San Diego epigenome Center**	http://epigenome.ucsd.edu/antibodies.html	histon modifications	101	56		7	5 member labs			Peptide dot blot		No	different lots are tested	
**SeekQuence**	http://www.seekquence.com/#!/	"select research products with confidence"	>2.48 M	19,660	84	110		x	x	84 differents applications: WB, IHC, FACS, IP, ELISA and others	x	soon	researcher can start on the “other side”, the publication side to search for Ab, sort for citations	
**The Human Protein Atlas**	http://www.proteinatlas.org	staining of human tissue	24,028	16975	human	40 suppliers and in-house generated antibodies (75)	control experiments are performed in consortium			IHC, protein array, IF, WB, adsorption	x	not at the moment	combination of transcriptomics analysis with antibody-based proteomics in human tissues and organs and provide the original immunohistochemistry-based images with details on the protein expression at a single-cell resolution.	
**Visabl**	http://www.visabl.com/	clinical diagnostic search machine			human	16			x	vendor dependent	x	No	different antibody panels (e.g. lung panel) have been compiled	

(Empty cells illustrate missing information. Either there was no answer to the survey or information is not easy accessible on the website.)
