# Supplementary material for: How to avoid pitfalls in antibody use
Source: F1000Res. 2015 Sep 7;4:691. [Version 1] doi: 10.12688/f1000research.6894.1 (PMC4722690; doi:10.12688/f1000research.6894.1)
Supplement: Peer review history

# Peer review: Referee response for version 1

**DOI:** 10.5256/f1000research.7423.r11018

**Published:** 2016-01-20

**Authors:** Anita Bandrowski

**Affiliations:** 1 University of California, San Diego, CA, USA

The report entitled "How to avoid pitfalls in antibody use" by Pauly and Hanack is a reasonable opinion piece with useful information and results from their survey. As such, it deserves indexing. I have already used the supplementary figure a couple of times as reference.

However, in my opinion, the piece does require a thorough tightening up in writing style. The authors should improve the writing to make the piece more formal to align with standard journal practices, removing most uses of "I", "we" and "my", strewn throughout the article.

For example:

current text "What is the target protein I want to characterize?"

*may read better as "What is the target protein to be characterized?"*

current text "How is my target protein structured in these samples?"

*may read better as "What is the configuration (concentrated protein, within complex tissue, membrane bound) of the target protein in the samples?"*

current text "Of course we agree with James Trimmer that “antibodies are not magic reagents” ^5^, but properly designed, characterized, validated and used, some can come close."

*may read better as "Indeed, James Trimmer the founder of NeuroMab states that “antibodies are not magic reagents” ^5^, but properly designed, characterized, validated and used, some can come close."*

The informal style detracts from the message and should be updated to increase impact.

For an example of masterful text, in this style please see:

http://www.sciencedirect.com/science/article/pii/S2212968516300022

I have read this submission. I believe that I have an appropriate level of expertise to confirm that it is of an acceptable scientific standard.

# Peer review: Referee response for version 1

**DOI:** 10.5256/f1000research.7423.r11019

**Published:** 2015-11-23

**Authors:** Simon Glerup Pedersen

**Affiliations:** 1 Department of Biomedicine, Aarhus University, Aarhus, Denmark

Paula and Hanack touch upon a very timely and important topic. Namely, the use of antibodies in research and how this affects scientific reproducibility. The authors intend to provide a guideline to how antibodies should be selected (or generated) before the onset of a scientific project.

This is an opinion article and therefore reflects the personal view of the authors. Although, I do not fully agree with all of the statements in the paper, I still consider this an interesting contribution to the discussion of how we can achieve a transparent use of research antibodies.

My main concern is their view on standardization of antibody validation. Antibodies may require very different protocol and buffer conditions in order to reach their optimal performance. One example is described by Ghatak *et al*. http://www.sciencedirect.com/science/article/pii/S2215016114000211. A standardized antibody validation system would completely miss such information. Instead, I consider a system for effective sharing of antibody performance details from scientists around the World. This would be much more effective for achieving a reproducible use of antibodies in research.

I have read this submission. I believe that I have an appropriate level of expertise to confirm that it is of an acceptable scientific standard.
